# Volumetric Trends Associated with MR-guided Stereotactic Laser Amygdalohippocampectomy in Mesial Temporal Lobe Epilepsy

**DOI:** 10.7759/cureus.2376

**Published:** 2018-03-27

**Authors:** Arthur Carminucci, Nitesh V Patel, Sri Sundararajan, Irwin Keller, Shabbar Danish

**Affiliations:** 1 Neurosurgery, Rutgers Robert Wood Johnson Medical School, Piscataway, USA; 2 Radiology, Rutgers Robert Wood Johnson Medical School, Piscataway, USA

**Keywords:** epilepsy, laser therapy, litt, laser ablation, slah

## Abstract

Objective: Magnetic resonance (MR)-guided stereotactic laser amygdalohippocampectomy is a minimally invasive procedure for the treatment of refractory epilepsy in patients with mesial temporal sclerosis. Limited data exist on post-ablation volumetric trends associated with the procedure.

Methods: 10 patients with mesial temporal sclerosis underwent MR-guided stereotactic laser amygdalohippocampectomy. Three independent raters computed ablation volumes at the following time points: pre-ablation (PreA), immediate post-ablation (IPA), 24 hours post-ablation (24PA), first follow-up post-ablation (FPA), and greater than three months follow-up post-ablation (>3MPA), using OsiriX DICOM Viewer (Pixmeo, Bernex, Switzerland). Statistical trends in post-ablation volumes were determined for the time points.

Results: MR-guided stereotactic laser amygdalohippocampectomy produces a rapid rise and distinct peak in post-ablation volume immediately following the procedure. IPA volumes are significantly higher than all other time points. Comparing individual time points within each raters dataset (intra-rater), a significant difference was seen between the IPA time point and all others. There was no statistical difference between the 24PA, FPA, and >3MPA time points. A correlation analysis demonstrated the strongest correlations at the 24PA (r=0.97), FPA (r=0.95), and 3MPA time points (r=0.99), with a weaker correlation at IPA (r=0.92).

Conclusion: MR-guided stereotactic laser amygdalohippocampectomy produces a maximal increase in post-ablation volume immediately following the procedure, which decreases and stabilizes at 24 hours post-procedure and beyond three months follow-up. Based on the correlation analysis, the lower inter-rater reliability at the IPA time point suggests it may be less accurate to assess volume at this time point. We recommend post-ablation volume assessments be made at least 24 hours post-selective ablation of the amygdalohippocampal complex (SLAH).

## Introduction

Surgical resection is considered the “gold standard” for the treatment of medically refractory mesial temporal lobe epilepsy (MTLE). The most common surgical treatments include anterior temporal lobectomy (ATL) and selective amygdalohippocampectomy (SAH) [1]. Patients undergoing ATL achieve seizure-free outcomes, ranging from 60%-80% [2-3]. While these results are effective, they do not come without potentially significant morbidity to the patient. These side effects involve impairments in neurocognitive functioning, including deficits in naming and verbal memory when the procedure is performed in the dominant hemisphere [4]. Despite the proven benefits of surgery, it remains vastly underutilized by patients who are potential candidates. Barriers to surgery include patients’ fear of invasive surgery, potential side effects, and the misconception that surgery should be considered last-resort treatment [5].

More selective stereotactic procedures have emerged that may alleviate some of the potential side effects of open surgery. Magnetic resonance imaging (MRI)-guided laser-induced thermal therapy (MRgLITT) is a minimally invasive laser-based surgical approach for the ablation of tissue through a fiber optic catheter. Focused light energy (photons), emitted through a cooled catheter, is converted to thermal energy in target tissues. Real-time MRI monitoring allows for the accurate ablation of target structures while limiting damage to surrounding tissue [6]. The application of MRgLITT has spanned from the treatment of systemic tumors, primary and secondary intracranial tumors, to chronic pain [7-14]. This procedure has been recently applied to the selective ablation of the amygdalohippocampal complex (SLAH) in patients with intractable MTLE. Early case series have shown promising results [15-17]. While there are no clinical studies to date directly comparing the two, emerging evidence may support SLAH as a viable, minimally invasive alternative to ATL [18].

As utilization of MRgLITT in epilepsy surgery increases, a better understanding of the changes in post-ablation volumes over time will be necessary to understand and guide post-operative clinical management. We have recently reported a method to precisely quantify post-ablation lesional volume in patients undergoing MRgLITT for intracranial neoplasms [19]. In this study, we apply our established methodology for post-ablation volume quantification to patients receiving MRgLITT of the amygdalohippocampal complex in mesial temporal sclerosis. The primary goal of this work is to assess volumetric trends in the ablation area over time in patients undergoing SLAH in mesial temporal sclerosis and to understand the time point at which this measurement can occur with the least inter-rater variability. This will be important, as future studies will require a multi-institutional approach.

## Materials and methods

Patient selection

The study was conducted with the approval of our institutional review board (Pro0220110296) and represents a retrospective cohort study that used data from a prospectively accumulated database. All patients undergoing SLAH at our institution were reviewed. Patient demographics where recorded. Patients had MRI imaging at various time points, including pre-ablation (PreA), immediate post-ablation (IPA), 24 hours post-ablation (24PA), first follow-up post-ablation (FPA) between one and three months, and greater than three months follow-up post-ablation (>3MPA).

Surgical procedure

We have described this procedure in detail in our prior studies [6]. In brief, skull pins are utilized for computed tomography (CT)-based registration and is then merged with a preoperative planning MRI using Medtronic Stealth S7 (Medtronic, Inc., Minneapolis, MN, US). The entry site and target trajectory for the amygdalohippocampal complex are determined. The patient is positioned prone and the head is secured with a Mayfield head holder. A stab incision is made at the entry site and a burr hole is created using a 3.2-mm handheld drill. The Visualase Thermal Therapy System (Medtronic, Inc., Minneapolis, MN, US) bone anchor is then secured to the skull using the alignment rod and precision aiming device (PAD). The laser catheter (Visualase, Inc., 15-W, 980-nm diode laser, flexible diffusing tipped fiber optic, and 17-gauge internally cooled catheter) is passed through the bone anchor to the depth previously determined during stereotactic planning to reach the target site.

The patient is transferred to a diagnostic MRI suite (Ge Excite, GE, Massachusetts, US, 1.5 Tesla) to perform the ablation procedure. The Visualase software is used to set thermal safety margins with maximum ablation temperature (90°C) at the center of the target lesion and maximum ablation temperature (50°C) at the target periphery to protect the surrounding tissue. The laser ablation procedure is then administered at the appropriate dose and duration to achieve the maximal safe ablation of the amygdalohippocampal complex, as determined by the surgeon.

Volume determination

Ablation volumes were measured by three independent raters (two neurosurgeons and one radiologist) experienced in the LITT procedure and intra- and postoperative radiographic results. Volumes were determined using OsiriX DICOM Viewer (Pixmeo, Bernex, Switzerland). Each rater was blinded to the volume measurements obtained by the other two raters. Five time points were selected for volume determination; pre-ablation (PreA), immediate post-ablation (IPA), 24 hours post-ablation (24PA), first follow-up post-ablation (FPA) between one and three months, and greater than three months follow-up post-ablation (>3MPA). For preA, an axial T2-weighted MRI sequence was used for volume analysis. For the remainder of the time points, the ablation area on post-contrasted imaging was defined by the hypointense necrotic center surrounded by a thin rim of hyperintense enhancement.

All ablation volumes were measured using OsiriX. Patient MRIs were downloaded from our institution’s picture archiving and communication system (PACS) system and uploaded into the OsiriX library. The “draw” function of the OsiriX software was used to trace the region of interest (ROI) on each slice of the MRI sequence containing an area of ablation. The trace-line thickness was set to 1/6th of the inherent OsiriX maximal line thickness [19]. The total ablation volume was determined by the “Compute ROI Volume” function, which fuses all the given ROIs to the approximate volume.

Computational and statistical analysis

Comparisons were made between the three raters’ datasets. The statistical analysis consisted of descriptive and comparative statistics using Microsoft Excel (Microsoft, Inc., Redford, WA) and SPSS (IBM, Inc., Armonk, NY). Descriptive statistics were derived from each individual rater’s dataset. Inter-rater reliability was assessed using both the student’s t-test and analysis of variance (ANOVA) across all three raters’ datasets.

## Results

Patient demographics

A total of 10 patients underwent the SLAH procedure. The subjects’ demographical data is summarized in Table 1. The mean ablation power was 10.39+/-1.10 W and the mean ablation time was 9.03 +/- 3.61 minutes (Table 2).

**Table 1:** Patient Demographics MTS: mesial temporal sclerosis

Patient	Age	Gender	Diagnosis
A	61	Male	Left MTS
B	27	Female	Right MTS
C	28	Male	Right MTS
D	50	Male	Left MTS
E	31	Female	Left MTS
F	23	Male	Left MTS
G	21	Female	Right MTS
H	55	Female	Left MTS
I	36	Female	Right MTS
J	53	Male	Right MTS

**Table 2:** Ablation Parameters w: watts m: minutes

Patient	Average Power (w)	Ablation Time (m)
A	10	12
B	9	5.52
C	10	6.15
D	10	7.11
E	10.2	15.36
F	12.7	7.3
G	12	5
H	10	11.39
I	10	7.30
J	10	13.10
Mean	10.39 +/- 1.10	9.03 +/- 3.61

Intra-rater comparisons

**Figure 1:**
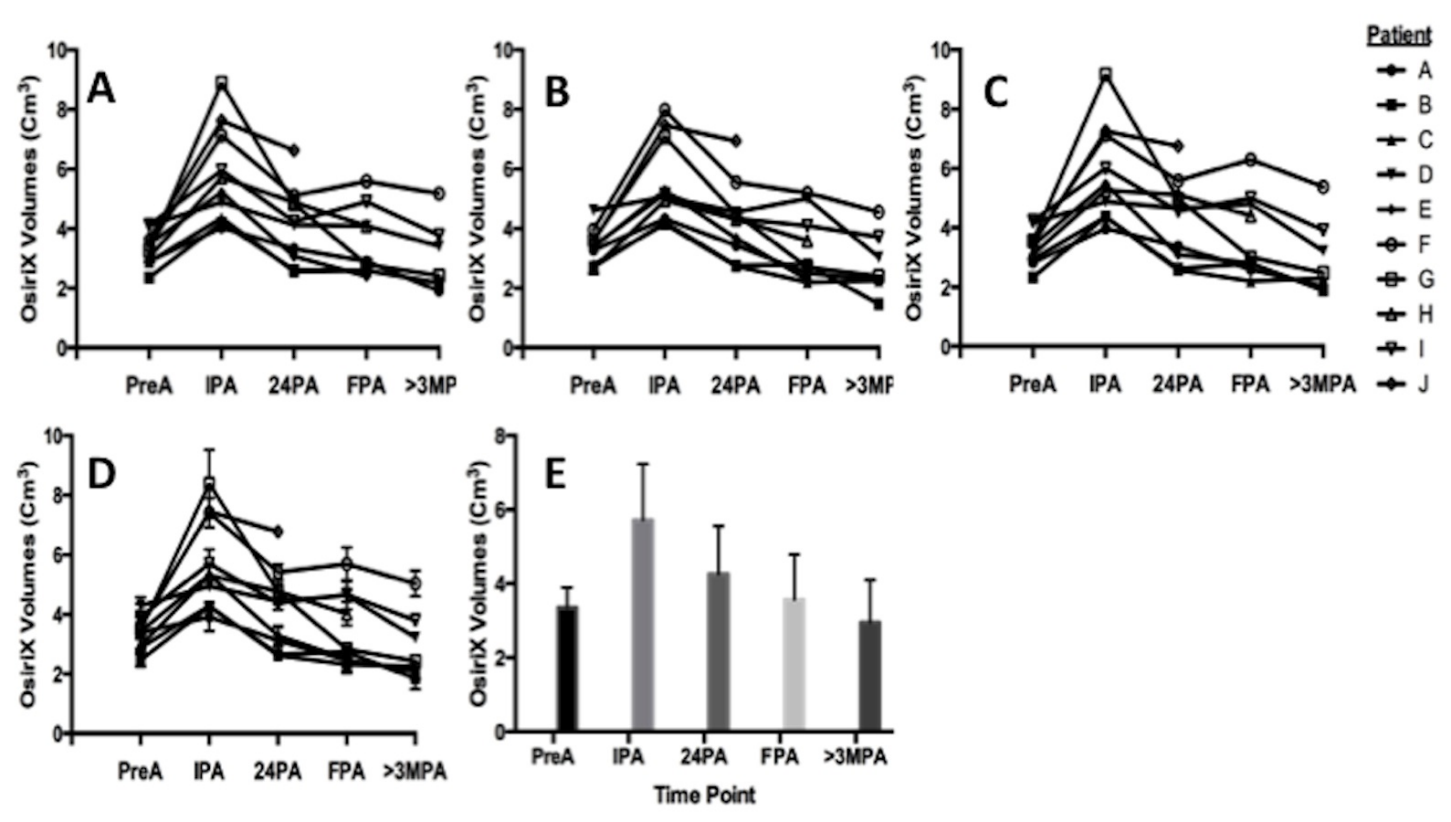
Volumetric Trend in Post-operative Ablation Volume Volumetric trends over time for 10 patients undergoing MRgLITT for MTS. The volume measurements (y-axis) for each individual patient is plotted over time. The X-axis contains the chosen time points, pre-ablation (PreA), immediate post-ablation (IPA), 24 hours post-ablation (24PA), first follow-up post-ablation (FPA) between one and three months, and greater than three months follow-up post-ablation (>3MPA), for volume determination. A: change in volume over time for each patient, as determined by Rater 1. B, C: volume measurements determined by Raters 2 and 3, respectively. D: Demonstrates the average volume combining all three rater’s datasets. E: Bar graph depicting trends in volume over time using the combined rater dataset. MRgLITT: MRI-guided laser-induced thermal therapy

Figure 1 demonstrates the volumetric trend in post-operative ablation volume over time for each patient. Following ablation, all subjects experienced a distinct peak in post-ablation volume at the IPA time point. Following the IPA time point, volumes trended downward over time across the remaining time points.

Table 3 summarizes the student’s t-test analysis between time points for raters’ 1, 2, and 3. The IPA volume was significantly higher than all other time points (PreA, 24PA, FPA, and >3MPA). This finding was consistent among all raters. Furthermore, comparisons between the remaining 24PA, FPA, and >3MPA showed no statistical differences, with the exception of the 24PA vs >3MPA for rater 2 (p=0.024) (See Appendix for intra-rater comparisons (complete set)).

**Table 3:** Intra-rater Comparisons pre-ablation (PreA), immediate post-ablation (IPA), 24 hours post-ablation (24PA)

Intra-Rater Student's t-Tests					
Rater 1		Rater 2		Rater 3	
IPA vs. PreA	P < 0.001	IPA vs. PreA	P < 0.001	IPA vs. PreA	P < 0.001
IPA vs. 24PA	P = 0.02	IPA vs. 24PA	P = 0.047	IPA vs. 24PA	P = 0.045
IPA vs. 1-3mo	P = 0.003	IPA vs. 1-3mo	P = 0.002	IPA vs. 1-3mo	P = 0.01
IPA vs 3+mo	P = 0.001	IPA vs 3+mo	P = 0.001	IPA vs 3+mo	P = 0.002

Inter-rater comparisons

Table 4 shows the results of ANOVA testing to gauge inter-rater reliability. There was no significant difference found among the three raters’ datasets. Table 5 shows the results of Pearson correlation between raters in head-to-head style comparisons. All correlations were strong; however, the correlation coefficient at PreA and IPA was lower than those values at all other time points. Specifically, at PreA, Rater 2 vs. 1 and Rater 1 vs. 3 showed a lower correlation coefficient. At IPA, the same result was seen.

**Table 4:** Inter-rater Comparison f-Value: variance pre-ablation (PreA), immediate post-ablation (IPA), 24 hours post-ablation (24PA)

Inter-Rater Reliability ANOVA					
	PreA	IPA	24PA	1-3 Mo	3+
f-Value	0.23	0.85	1.92	3.4	1.52
p-Value	0.79	0.44	0.17	0.06	0.25

**Table 5:** Pearson’s Correlation Coefficients by Rater and Time Point pre-ablation (PreA), immediate post-ablation (IPA), 24 hours post-ablation (24PA)

Pearson Correlation					
	PreA	IPA	24PA	1-3 Mo	3+
Rater 1 vs Rater 2	0.75	0.89	0.96	0.91	0.97
Rater 2 vs Rater 3	0.97	0.99	0.99	0.97	1.00
Rater 2 vs Rater 3	0.75	0.87	0.96	0.96	0.97
Average	0..82	0.92	0.97	0.95	0.99

Radiographic features

**Figure 2:**
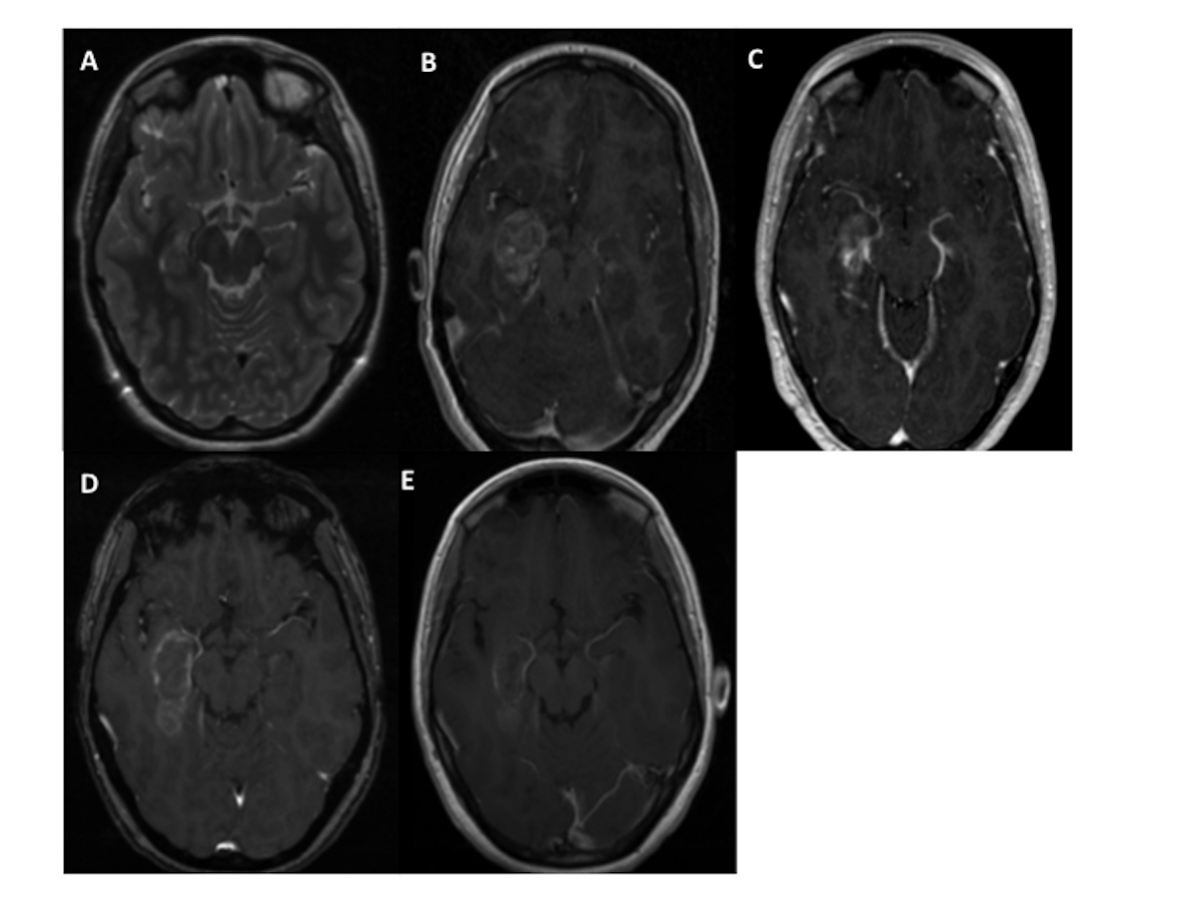
Radiographic Characteristic of the Ablation Cavity over Time A: Pre-operative T2 sequence MRI for patient G, showing right-sided mesial temporal sclerosis. B: IPA time point, with strong contrast enhancement. C, D: 24PA and FPA time point respectively, ring enhancement persists. E: >3MPA time point demonstrating decreased contrast enhancement and beginnings of cavitation at the center of ablation bed. Immediate post-ablation (IPA), 24 hours post-ablation (24PA), first follow-up post-ablation (FPA), and greater then 3 months follow-up post-ablation (>3MPA)

The radiographic characteristic of the ablation cavity at each time point for a single patient is illustrated in Figure 2. Immediately following the ablation there is an uptake of gadolinium enhancement throughout the ablation bed. A ring of enhancement demarcates the boundaries of the cavity. Additionally, there is edema and contrast extravasation surrounding the cavity. At the 24PA and FPA time points, contrast enhancement is evident with decreasing edema and contrast extravasation. By the >3MPA, decreased contrast enhancement is noticed along with the development of gliosis at the center of the cavity. Similar observations at each time point were seen for all patients in the study.

## Discussion

SLAH is a potential alternative to anterior temporal lobectomy. Willie et al. treated 13 patients with mesial temporal lobe epilepsy with MRgLITT. In their study, 67% of patients achieved seizure freedom and overall 77% of patients achieved meaningful clinical improvement [16]. Similar initial reports of SLAH have shown efficacious clinical results approaching those of anterior temporal lobectomy [20,15,17]. There are several advantages of SLAH compared to open resection. ATL can result in neurocognitive deficits, including language and verbal memory impartment due to damage to surrounding critical structures during resection [4,13,21]. SLAH is a minimally invasive, stereotactic procedure that allows for real-time monitoring of the thermal ablation zone, thus confirming the ablation target and safely sparing adjacent structures. Laser ablation in temporal lobe epilepsy results in improved cognitive outcomes, particularly naming and recognition [22]. Additionally, SLAH is associated with short hospital stays and low rate of postoperative morbidity [10,16-17,23]. While there are no current randomized, controlled clinical studies directly using SLAH with ATL, a decision analysis based on early clinical data suggests that laser ablation has similar utility to open surgery [18].

As the utilization of SLAH for the treatment of medically refractory epilepsy increases, a better understanding of post-procedure assessments will be necessary to guide clinical decision-making. Currently, SLAH is assessed through clinical parameters, whether or not a patient obtains seizure freedom. Limited data exist for treatment paradigms for patients who fail to obtain seizure control following initial laser ablation. Guidelines on whether to repeat an ablation or proceed to open surgery tend to be based on institutional experiences and patient preferences. There are no current, standardized quantitative assessments of the procedure. Quantitative information regarding SLAH can be used to assess whether or not an adequate ablation volume was obtained and if a repeat ablation should be performed in patients who fail to achieve seizure freedom.

In this study, we performed a quantitative assessment of the dynamics of ablation volumes over time. In all 10 patients following the LITT procedure, the ablation volume rapidly reached a peak volume at the IPA time point. As ablation volumes were trended over time, volumes gradually began to decrease and stabilize. The IPA volume was significantly larger than any other time point measured, indicating a maximal increase in ablation volume immediately following the procedure. Following the acute peak in volume at IPA, volumes graphically trended downward over time (Figure 1), although there was no significant difference between the 24PA, FPA, and >3MPA time points. A similar pattern in the rise and peak of lesion volume at IPA is seen in patients undergoing LITT for intracranial tumors [19].

To improve the accuracy of our volume measurements, MRI sequences were analyzed by three independent raters (two neurosurgeons and one radiologist). All three raters were familiar with the SLAH procedure and experienced in using the OsiriX software. Raters were blinded to patient and time point in order to reduce inter-rater bias. Comparing individual time points within each rater’s dataset, a significant difference was seen between the IPA time point and all other time points. This further supports the time-dependent variability of post-ablation volumes. Of note, there was no statistical difference between the 24PA, 1-3mo, and 3+mo time points within each raters' data set, except for Rater 2's assessment of 24PA vs. >3MPA (p=0.024, Appendix, Table 6). This discrepancy was not observed in Rater 1's or Rater 3’s data set. Through a correlation analysis, inter-rater reliability was high among all three raters across all time points. The strongest correlations were demonstrated at the 24PA (r=0.97), FPA (r=0.95), and >3MPA time points (r=0.99). The IPA time point demonstrated a weaker correlation coefficient, r=0.92. The lower inter-rater reliability at the IPA time point suggests it may be less accurate to assess volume at this time point. The increased variability at IPA is likely attributed to the transient disruption of the blood-brain barrier immediately following the ablation. The heterogeneity of gadolinium dispersion into the ablation bed is secondary to the disruption of vascular integrity. A leakage of contrast from injured capillaries leads to poorly delineated lesion borders at this time point. As the blood-brain barrier reconstitutes over time, there is a more clearly delineated contrast-enhancing border seen at the subsequent time points, which may be a more accurate representation of the ablation zone. However, note that the only way to really understand the true ablation zone is to examine the tissue pathologically, which is not possible. Additionally, there was an unexpected weaker correlation at the PreA time point. Since no ablation has been performed at this time point, there is no surrounding ring of gadolinium enhancement measured. We chose to use the T2-weighted MRI sequences at the PreA time point to help delineate the borders of the amygdalohippocampal complex.

Few studies have looked at a volume-based analysis SLAH in temporal lobe epilepsy. Kang et al. performed a volumetric analysis on 16 patients undergoing SLAH for mesial temporal lobe epilepsy by calculating ablation volume between the pre-op MRI sequence and the immediate post-procedure MRI sequence. Their study found no differences in the total ablation volume between seizure-free and non-seizure-free patients [17]. Similarly, Willie et al. compared pre-and post-post procedural volumes and found no correlation between total ablation volume and seizure freedom [16]. Our study has shown time-dependent variability in post-ablation volume measurements. Given the variability of the initial, immediate post-ablation volume compared to subsequent chronologic volume measurements, quantitative correlations with clinical outcomes should be made at least 24 hours post-ablation.

Atsina et al. described the longitudinal MRI characteristics of LITT for epilepsy. They report the development of a ring-enhancing lesion at the site of ablation immediately following the procedure. The ablation cavity transitioned from persistent enhancement with mild edema in the subacute phase to a gliotic cavity with minimal to no enhancement in the chronic phase [24]. Similar radiographic characteristics were observed in our patient population, as illustrated in Figure 2.

As with any retrospective study, several factors limit this study. First, we reviewed a relatively small sample population. As SLAH is a relatively new procedure for temporal lobe epilepsy, and only performed at a handful of institutions, it is difficult to amass a large population of patients to study. Additionally, the time points chosen for analysis were limited by the availability of MRI studies for each patient. While all patients had a PreA, IPA, and 24PA MRI, two patients did not have a >3MPA scan and one patient did not have an FPA and >3MPA. The availability of patient MRI scans was affected by patient follow-up and time of initial procedure compared to when this analysis was performed. However, as the greatest variability in observed volume occurs at the IPA time point, the exclusion of just a few follow-up scans would not appear to affect the overall outcome of the study.

It is quite possible that a larger subset would reveal a significant difference between time points after IPA. It is important to realize that the post-ablation volume is a moving target and that as a field, we must come to an agreement on the time point in which we make a determination of the post-ablation volume, especially if we are going to attempt to correlate it with clinical outcomes. Certainly, this will be necessary to compare data across institutions as multi-center studies are developed.

## Conclusions

The response of tissue deformation following SLAH for stereotactic amygdalohippocampectomy ablation is dynamic. The maximal increase in post-ablation volume is rapidly achieved immediately following the procedure, which decreases at 24 hours post-procedure and stabilizes over time. The variability in ablation volume at the IPA time point questions the accuracy of measuring lesion volumes at this time point.

We recommend post-ablation volume assessments be made at least 24 hours post-SLAH, when lesion size has stabilized. Larger prospective series are needed to correlate post-ablation volume with seizure freedom.

## Appendices

**Table 6:** Intra-rater Comparisons (Complete Set) pre-ablation (PreA), immediate post-ablation (IPA), 24 hours post-ablation (24PA)

Intra-Rater Student's t-Tests					
Rater 1		Rater 2		Rater 3	
IPA vs. PreA	P < 0.001	IPA vs. PreA	P < 0.001	IPA vs. PreA	P < 0.001
IPA vs. 24PA	P = 0.02	IPA vs. 24PA	P = 0.047	IPA vs. 24PA	P = 0.045
IPA vs. 1-3mo	P = 0.003	IPA vs. 1-3mo	P = 0.002	IPA vs. 1-3mo	P = 0.01
IPA vs 3+mo	P = 0.001	IPA vs 3+mo	P = 0.001	IPA vs 3+mo	P = 0.002
24PA vs. PreA	P = 0.083	24PA vs. PreA	P = 0.064	24PA vs. PreA	P = 0.054
1-3mo vs. PreA	P = 0.568	1-3mo vs. PreA	P = 0.951	1-3mo vs. PreA	P = 0.418
1-3mo vs. 24PA	P = 0.325	1-3mo vs. 24PA	P = 0.124	1-3mo vs. 24PA	P = 0.378
3+mo vs. PreA	P = 0.422	3+mo vs. PreA	P = 0.162	3+mo vs. PreA	P = 0.479
3+mo vs. 24PA	P = 0.078	3+mo vs. 24PA	P = 0.024	3+mo vs. 24PA	P = 0.066
3+mo vs. 1-3mo	P = 0.334	3+mo vs. 1-3mo	P = 0.336	3+mo vs. 1-3mo	P = 0.299
